# Erratum

**DOI:** 10.1590/1980-549720220014.supl.1erratum

**Published:** 2024-09-30

https://doi.org/10.1590/1980-549720220014.supl.1erratum

In the manuscript “**Trend analysis of lung cancer incidence and mortality in Grande Cuiabá, Mato Grosso, Brazil, 2000 to 2016**”, DOI: https://doi.org/10.1590/1980-549720220014.supl.1, published in the Rev Bras Epidemiol 2022; 25: e220014.supl.1:

**On page 1 it was included:**

ASSOCIATED EDITORS: Elisete Duarte http://orcid.org/0000-0002-0501-0190, Gulnar Azevedo e Silva http://orcid.org/0000-0001-8734-2799

SCIENTIFIC EDITOR: Cassia Maria Buchalla http://orcid.org/0000-0001-5169-5533

